# Genomic Analysis of *Talaromyces verruculosus* SJ9: An Efficient Tetracycline-, Enrofloxacin-, and Tylosin-Degrading Fungus

**DOI:** 10.3390/genes15121643

**Published:** 2024-12-21

**Authors:** Jing Fu, Xiaoqing Wu, Chi Zhang, Yuhan Tang, Fangyuan Zhou, Xinjian Zhang, Susu Fan

**Affiliations:** 1 Shandong Provincial Key Laboratory of Applied Microbiology, Ecology Institute, Qilu University of Technology (Shandong Academy of Sciences), Jinan 250103, China; 10431221210@stu.qlu.edu.cn (J.F.); 10431221165@stu.qlu.edu.cn (C.Z.); 10431221166@stu.qlu.edu.cn (Y.T.); 2 Institute of Ecology, Shandong Academy of Sciences, Jinan 250103, China; wxq@qlu.edu.cn (X.W.); fangyuan_zhou@qlu.edu.cn (F.Z.)

**Keywords:** whole-genome sequencing, antibiotic degradation, *T. verruculosus*, functional annotation

## Abstract

Background/Objectives: Many fungi related to *Talaromyces verruculosus* can degrade a wide range of pollutants and are widely distributed globally. *T. verruculosus* SJ9 was enriched from fresh strawberry inter-root soil to yield fungi capable of degrading tetracycline, enrofloxacin, and tylosin. Methods: *T. verruculosus* SJ9 genome was sequenced, assembled, and annotated in this study utilizing bioinformatics software, PacBio, and the Illumina NovaSeq PE150 technology. Results: The genome size is 40.6 Mb, the N50 scaffold size is 4,534,389 bp, and the predicted number of coding genes is 8171. The *T. verruculosus* TS63-9 genome has the highest resemblance to the *T. verruculosus* SJ9 genome, according to a comparative genomic analysis of seven species. In addition, we annotated many genes encoding antibiotic-degrading enzymes in *T. verruculosus* SJ9 through genomic databases, which also provided strong evidence for its ability to degrade antibiotics. Conclusions: Through the correlation analysis of the whole-genome data of *T. verruculosus* SJ9, we identified a number of genes capable of encoding antibiotic-degrading enzymes in its gene function annotation database. These antibiotic-related enzymes provide some evidence that *T. verruculosus* SJ9 can degrade fluoroquinolone antibiotics, tetracycline antibiotics, and macrolide antibiotics. In summary, the complete genome sequence of *T. verruculosus* SJ9 has now been published, and this resource constitutes a significant dataset that will inform forthcoming transcriptomic, proteomic, and metabolic investigations of this fungal species. In addition, genomic studies of other filamentous fungi can utilize it as a reference. Thanks to the discoveries made in this study, the future application of this fungus in industrial production will be more rapid.

## 1. Introduction

Secondary metabolites having anti-pathogenic qualities that are produced by microorganisms and higher plants are known as antibiotics [1,2]. Over the past several decades, there has been a sharp increase in the consumption of antibiotics [3]. However, antibiotics are not completely absorbed by organisms and are eventually excreted through the feces and urine at a rate of 28–88%, resulting in high residual levels of antibiotics in animal feces [4]. With the widespread use of animal waste in agricultural soil, antibiotics also infiltrate the soil via livestock and poultry manure [5,6]. Soil has become a common “sink” for the widespread detection of various antibiotics in the environment [7,8].

The emergence of bioremediation technology has brought new ideas and approaches to the management of soil antibiotic contamination. Fungi have several potential benefits in persistent organic pollutant degradation since they are more dynamic and tolerant to harsh conditions than bacteria [9]. Some fungi have been shown to break down antibiotic drugs. Ren et al. found that *Penicillium oxalicum* strain *P. oxalicum* RJJ-2 removed 84.88% of erythromycin in 96 h [10]. Erythromycin can be degraded by the enzyme-catalyzed breaking of glycosidic bonds by *Curvularia* sp. RJJ-5 strain [11]. Ahumada-Rudolph et al. screened and isolated five strains with the ability to degrade hygromycin in sediment samples from a salmon farming area in a fjord in southern Chile, and they were identified as *Trichoderma harzianum*, *Trichoderma deliquescens*, *Penicillium crustosum*, *Rhodotorula mucilaginosa*, and *Talaromyces atroroseus*. Their study showed that all strains exhibited a significant decrease in concentration in the first 2 d of exposure to hygromycin, and the degradation rates in response to 21 d of hygromycin at 250 mg/L were 92%, 85%, 83%, 73%, and 72%, demonstrating that marine fungi obtained from marine sediments are capable of degrading tetracycline antibiotics [12]. Lucas et al. treated wastewater with *Trametes versicolor* ATCC42530 and removed 77% of 47 antibiotics of seven different categories (such as fluoroquinolones, tetracyclines, and sulfonamides), which was significantly greater than the rates of traditional treatments [13]. Antibiotic microbial remediation strategies currently in use are often based on bacteria or their degradable metabolites and secretions, such as microflora [14], degrading bacteria [15], degrading enzymes [16,17], and degrading genes [18,19]. Among them, degradative enzymes remove antibiotics with higher specificity and maneuverability; this is an antibiotic degradation technique that is more cost-effective and environmentally friendly. The antibiotic-degrading enzymes studied so far are mainly derived from antibiotic-resistant bacteria. For example, lignin peroxidase (LiP), manganese peroxidase (MnP), intracellular cytochrome P450 (CYP450), and laccase (Lac) produced by white-rot fungi have the ability to degrade antibiotics such as TCs, FQs, and SAs [20,21,22,23]. Sun et al. used MnP from *Phanerochaete chrysosporium* to biodegrade tetracycline; the fungus was able to remove 80% of tetracycline within 3 h [24]. According to Becker et al., immobilizing Lac using *T. versicolor* could remove >70% of tetracycline within 24 h [25]. Lac can use oxygen as an electron acceptor to oxidize the substrate and then perform single-electron transfer; MnP can oxidize Mn^2+^ to Mn^3+^, chelate with organic acid, and then undergo an oxidation reaction to transform pollutants [16,26]. The multifunctional peroxidase in *Bjerkandera adusta* degrades sulfamethoxazole to produce the stabilized products 3A5MI, carboxylic acids (acetic acid and oxalic acid), and anions (nitrate, nitrite, and sulfate), suggesting that the aniline ring has been effectively degraded [27]. Glycosyltransferasewhich cloned from *Streptomyces lividans*, changes the structure of macrolide antibiotics via glycosylation, rendering them inactive [28]. Phosphotransferases found in Gram-negative bacteria, such as *Escherichia coli*, *Shigella*, *Vibrio*, and *Pasteurella*, and Gram-positive bacteria, such as *Bacillus* and *Staphylococcus*, can covalently modify macrolide antibiotics through prephosphorylation, thereby inhibiting the antibiotic’s binding to the ribosome and rendering it devitalized [29,30,31]. Degradative enzymes are an environmentally friendly, safe, and effective biodegradation method, and it is extremely important to explore more efficient degradation genes and degradative enzymes for the biodegradation of antibiotics in the environment.

In our recent study, a strain of *T. verruculosus* SJ9, which can effectively degrade tetracycline, enrofloxacin, and tylosin, was isolated from strawberry rhizosphere soil. The whole-genome sequence and database annotation data of *T. verruculosus* SJ9 are presented in this study. The purpose of this work was to theoretically support and furnish dependable reference data for future investigations into the mechanism of antibiotic degradation, transcriptomics, proteomics, metabolomics, and RNA genomes of *T. verruculosus* SJ9.

## 2. Materials and Methods

### 2.1. Procedures for Isolating and Identifying T. verruculosus SJ9

Strawberry rhizosphere soil samples were collected in Jinan City, Shandong Province, China. A total of 5 g soil was mixed with 50 mL of 0.9% sterile saline solution and shaken at a constant temperature of 28 °C and 200 r/min for 2 h. We added 5 mL of the supernatant to 25 mL of inorganic salt screening medium with 20 mg/L enrofloxacin. The culture solution was incubated at 28 °C and protected from light for 24 h. The above steps were repeated at 24-h intervals, and the concentration of enrofloxacin was increased at a gradient of 20 mg/L to reach a concentration of 200 mg/L in the culture solution. The culture medium supernatant was collected after domestication in sterile water and then diluted in culture medium at 10^−1^, 10^−2^, 10^−3^, 10^−4^, 10^−5^, 10^−6^, 10^−7^. Then, 100 μL of the diluted supernatant was added to potato dextrose agar (PDA) medium, and single colonies of different morphologies were picked and streaked on the plate for culture. The above steps were repeated, and the selected single colonies of degraded bacteria were stored at −80 °C. The functional strains of tetracycline and tylosin were isolated and purified using the same method as above.

With the aid of a DNA extraction kit, fungal genomic DNA was isolated from mycelium on PDA plates. The species name of the isolated strain was determined using a pair of fungal universal primers ITS1 (5′-TCCGTAGGTTAACCTGCGG-3′) and ITS4 (5′-TCCTCCGCTTATTGATATGC-3′). The recent relative sequences of the strains were searched in the NCBI database, and a phylogenetic tree was performed.

### 2.2. Biodegradation of Three Antibiotics

The concentrations of enrofloxacin, tetracycline, and tylosin were determined using high-performance liquid chromatography (HPLC, Agilent 1260, Santa Clara, CA, USA). The chromatographic column was an XTERRA RP C18 column (250 mm × 4.6 mm, 5 μm; Waters, Milford, MA, USA). Enrofloxacin and tetracycline were detected by the following method: the mobile phase was 60% 0.1% (*v*/*v*) formic acid aqueous solution and 30% pure acetonitrile. The mobile phase consisted of 20% (*v*/*v*) 0.1% formic acid and 80% (*v*/*v*) acetonitrile at a flow rate of 1 mL/min. The injection volume was 5 μL and the detection wavelength was 280 nm. The detection method for tetracycline was as follows: the mobile phase consisted of 20% (*v*/*v*) 0.1% formic acid and 80% (*v*/*v*) acetonitrile. The injection volume was 5 μL and the detection wavelength was 280 nm. The degradation rates of the three antibiotics were calculated as follows:

Degradation rate (%) = (Ct (CK) – Ct (T))/(Ct (CK)) × 100%.

where Ct (CK) represents the average concentration of antibiotics in the control group (CK) measured on day (time) t, and Ct (T) represents the average concentration of antibiotics in the experimental group (T) measured at the same time.

### 2.3. Extraction of Genome DNA, Library Construction, Sequencing, and Assembly

Initially, pure cultures of *T. verruculosus* SJ9 were inoculated onto PDA plates and incubated at 28 °C for 3 d. Subsequently, before being submitted to Beijing Novogene Bioinformatics Technology Co., Ltd. (Beijing, China) for DNA extraction and the development of the *T. verruculosus* SJ9 strain’s whole-genome sequence, fresh mycelium samples were gathered and quickly frozen in liquid nitrogen.

The genomic DNA of the samples was extracted using the GP1 approach, and then the DNA purity and integrity were evaluated using agarose gel electrophoresis and Qubit quantification. Subsequently, the SMRT Bell™ Template kit (version 2.0) [32] was used to construct the SMRT Bell Library. The NEBNext^®^ UltraTM DNA Library Prep Kit (NEB, Ipswich, MA, USA) was used to create a 350 bp, tiny, fragmented library [33]. Upon library qualification, whole-genome sequencing of *T. verruculosus* SJ9 was conducted at Beijing Novogene Bioinformatics Technology Co., Ltd., using the PacBio Sequel platform and Illumina NovaSeq PE150 (San Diego, CA, USA) [34]. SMRT Link (PacBio, Menlo Park, CA, USA) [35,36,37] was used for read assembly and to obtain exploratory results. Reads were compared to the constructed sequences after superfluous sequences from the first assembly results were eliminated. The genome’s repetitive sequences and GC bias were computed by manipulating the GC content and expanding the read depth of the assembly sequence, penalizing areas with assembly faults to produce the best possible assembly results [36].

### 2.4. Genomic Prediction and Genome Annotation

Several software techniques were utilized to determine the genome-encoded genes, repetitive sequences, and non-coding RNAs of *T. verruculosus* SJ9. First, protein-coding genes were obtained from the *T. verruculosus* SJ9 genome using Augustus (version 2.7) [38]. Subsequently, scattered repetitive sequences were predicted using RepeatMasker (Version open-4.0.5) [39], and tandem repeat sequences in DNA sequences were identified using Tandem Repeats Finder (Version 4.07b) [40]. Following that, transfer RNA (tRNA) genes were predicted using tRNAscan-SE (version 1.3.1) [41], and ribosomal RNA (rRNA) genes were predicted using rRNAmmer (version 1.2) [42] within the genome sequence. Small RNA (sRNA), small nuclear RNA (snRNA), and microRNA (miRNA) predictions were conducted using Rfam software to annotate the molecular level of these genes, according to data from the Rfam database. Then, the presence of sRNA, snRNA, and miRNA in the *T*. *verruculosus* SJ9 genome was confirmed using the cmsearch program in Rfam (version 1.1rc4) [43,44,45] with default parameters.

We utilized multiple databases to forecast gene functionality. These included the Gene Ontology (GO), Kyoto Encyclopedia of Genes and Genomes (KEGG), Clusters of Orthologous Groups (KOG), Non-Redundant Protein Database (NR), Transporter Classification Database (TCDB), P450, and Swiss-Prot. A whole-genome Blast search (E-value less than 1 × 10^−5^, minimal alignment length percentage larger than 40%) was performed against the above databases. The secretory proteins were predicted by the SignalP database [46]. Meanwhile, we analyzed the secondary metabolism gene clusters by the antiSMASH [47]. Circular genome data were graphed using Circos (0.69–9) [48]. In addition, we utilized the Pathogen Host Interactions (PHI) database and the Database of Fungal Virulence Factors (DFVF) to conduct analyses on pathogenicity and drug resistance. Additionally, the prediction of carbohydrate-active enzymes was carried out using the Carbohydrate-Active enZYmes Database.

The whole-genome sequence of strain *T. verruculosus* SJ9 has been deposited in GenBank under the accession number JBBVQN000000000. The raw sequencing data for the genome and the assembly reported in this paper are associated with NCBI BioProject: PRJNA1090279 and BioSample: SAMN40561778 within the GenBank. The SRA accession numbers are SRR29208833 and SRR31113075.

### 2.5. Annotation and PCR Validation of Antibiotic Degrading Enzymes

We searched for common antibiotic-degrading enzymes as well as annotated degrading enzyme-related genes from the gene function annotation database of strain *T. verruculosus* SJ9 and selected one gene from each degradation enzyme for PCR validation. The DNA was extracted in the same way as described above. One gene from each antibiotic degrading enzyme annotated in *T. verruculosus* SJ9 was then selected and the gene sequences were extracted. Primers were designed for PCR validation using Primer Primer 5.0 software. The PCR reaction system (50 μL) consisted of 2 μL DNA, 1.5 μL each of primer upstream and primer downstream, 25 μL KOD One^TM^ PCR Master Mix (TOYOBO, Shanghai, China), and 22 μL ddH_2_O. The PCR reaction was carried out under the following conditions: pre-denaturation at 94 °C for 2 min, denaturation at 98 °C for 10 s, annealing at 55 °C for 5 s, and then elongation at 68 °C for 40 s. A total of 35 cycles were carried out from denaturation to elongation. The final detection of PCR products was achieved with agarose gel electrophoresis.

### 2.6. Procedures and Tools in Comparative Genomic Analysis

*T. verruculosus* TS63-9, *T. marneffei Talaromyces marneffei* 11CN-20-091, *T. rugulosus Talaromyces rugulosus* W13939, *T. stipitatus Talaromyces stipitatus* ATCC 10500, *T. amestolkiae Talaromyces amestolkiae* CIB, *T. atroroseus Talaromyces atroroseus* IBT 11181, and *T. proteolyticus Talaromyces proteolyticus* PMI_201 were selected as reference genomes to be compared with the whole-genome data of *T. verruculosus* GXJ-SJ9 (Table S1). Genomic comparisons between representative and reference genomes were assessed using MUMmer (4.0.0candidate1) [49], and LASTZ (1.04.22) [50] was used for pairwise comparisons between *T. verruculosus* GXJ-SJ9 and seven reference genomes. Similar proteins were analyzed via rapid clustering using the Database for Highly Tolerant Clustering, with a 50% threshold for pairwise equivalence and a critical value of 0.7 for amino acid length differences [51]. Proteins from multiple target genomes were compared two-by-two using BLAST software [50] (Version 2.2.26) to filter the results where the comparison was not credible. Then, redundant data were eliminated using Solar (Version 0.9.6); the proteins were clustered according to the similarity of the comparisons using Hcluster-sg (Version 0.2.0) to obtain gene family clustering results. Evolutionary trees were constructed using PHYML (Version v3.0) (maximum likelihood); the bootstrap of homologous genes was set to 1000 [52]. Average nucleotide identity (ANI) was confirmed using FastANI (Version 1.34).

## 3. Results

### 3.1. Isolation and Characterization of T. verruculosus SJ9

**Figure 1:**
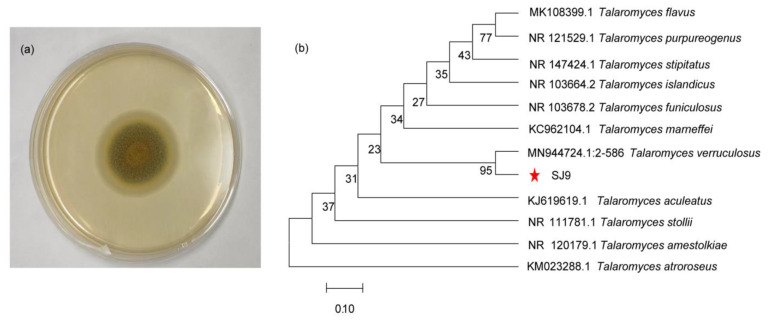
Morphological characteristics of *T. verruculosus* SJ9. (**a**) Colony morphology of strain SJ9 on PDA plate. (**b**) Identification of strain SJ9 based on phylogenomic analyses. The phylogenetic tree reveals that strain SJ9 is closely related to *T. verruculosus*. The red star indicates the SJ9 strain.

A total of 32 single colonies of culturable fungi were obtained via enrichment culture of fresh strawberry inter-root soil samples. We observed that strain SJ9 was enriched in three antibiotic media, namely enrofloxacin, tetracycline, and tylosin. Its colonies were round with a velvety texture; the mycelium was light orange-yellow with a pale orange-red center in the early stage, and then gradually turned into a greenish-gray color in the later stage (Figure 1a). The bacterium degraded enrofloxacin, tetracycline, and tylosin at an initial concentration of 200 mg/L by 70%, 48%, and 99%, respectively. The bacterium was able to degrade enrofloxacin, tetracycline, and tylosin within 10 d. The amplified internally transcribed spacer (ITS) sequences were compared using NCBI BLAST, and evolutionary tree phylogenetic analysis showed that strain SJ9 was most similar to MN944724.1:2-586 *T. verruculosus*. Based on its morphological characteristics, the strain was identified as *T. verruculosus* and named SJ9 (Figure 1b). In previous studies, *T. verruculosus* has also been found to degrade lignin [53] and polycyclic aromatic hydrocarbons [54] and produce tannase for tannin degradation [55]. *T. verruculosus* also can melt phosphorus and remediate cadmium contamination, as well as produce oxo-dienoic acid [56] and polysaccharide-degrading enzymes [57]; it is antimicrobial [58] and can remove theobromine [59].

### 3.2. Genome-Wide Analysis

#### 3.2.1. Genome Assembly and Characteristics of Genomic Analysis

The amount of reads in the raw offline information was collected from whole-genome analysis of *T. verruculosus* SJ9. It comprised 30 contigs, according to the preliminary assembly data, and the longest assembled contig measured 8,090,124 bp. The total contig length was 40,609,410 bp, while the N50 contig length was 4,534,389 bp. After aligning and evaluating the reads to the assembly sequence, the final assembly results revealed outcomes that were comparable to those observed in the preliminary assembly, and 45.4% of the constructed contig GC was present (Table 1).

#### 3.2.2. Gene Prediction

By analyzing the genomic data, it was predicted that there were 8171 coding genes in the genome of *T. verruculosus* SJ9, with a total length of 40,609,410 bp, which accounted for 27.72% of the total length of the genome, and the average length was 1378 bp (Table S2). LTR, DNA, LINE, SINE, RC, and Unknown were the six groups into which the DRs were divided. These categories made up 1.2317%, 0.309%, 0.4519%, 0.0073%, 0.0051%, and 0.0006% of the entire genome, with average lengths of 299, 207, 313, 58, 71, and 60 bp, respectively. With repeat lengths of 1~989 bp, 10~60 bp, and 2~6 bp, the tandem repeat sequences (TRs), minisatellite DNA, and microsatellite DNA were found for 0.6238%, 0.38%, and 0.05% of the entire genome, respectively (Table S3). Additionally, we observed RNAs in *T. verruculosus* SJ9, which are included in Table S4. tRNA (*n* = 124) was found to be the most abundant type of RNA, followed by snRNA (*n* = 8), sRNA (*n* = 2), and miRNA (*n* = 0).

### 3.3. Gene Functional Analysis

#### 3.3.1. Gene Functional Annotations

The protein sequences were forecasted by conducting a similarity analysis on 8171 coding genes from various public databases, including GO, KEGG, KOG, NR, TCDB, Pfam, CAZy, P450, Swiss-Prot, SignalP, PHI, and DFVF (Figure S1 and Table S5).

**Figure 2:**
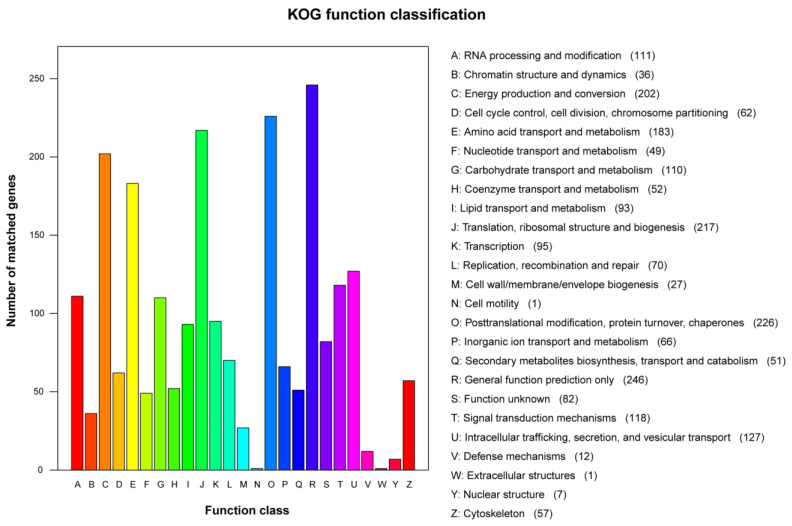
Clusters of orthologous groups of proteins (KOG) function classification of proteins in *T. verruculosus* SJ9. The x-axis shows the function of the class and the y-axis shows the number of matching genes. KOG functional classification is divided into 26 groups classified as A–Z, and each group has its own function. Different colors with their names and number of genes are also mentioned in the figure.

The orthologs of eukaryotes that are part of the COG database are stored in the KOG database. The statistical representation of all annotated genes within the KOG database is illustrated in Figure 2. The top four categories of KOG were “General function prediction only” (*n* = 246, 11.74%), “Posttranslational modification, protein turnover, chaperones” (*n* = 226, 10.79%), “Translation, ribosomal structure, and biogenesis” (*n* = 217, 10.36%), and “Energy production and conversion” (*n* = 202, 9.64%). These 25 groups included a total of 2095 genes.

**Figure 3:**
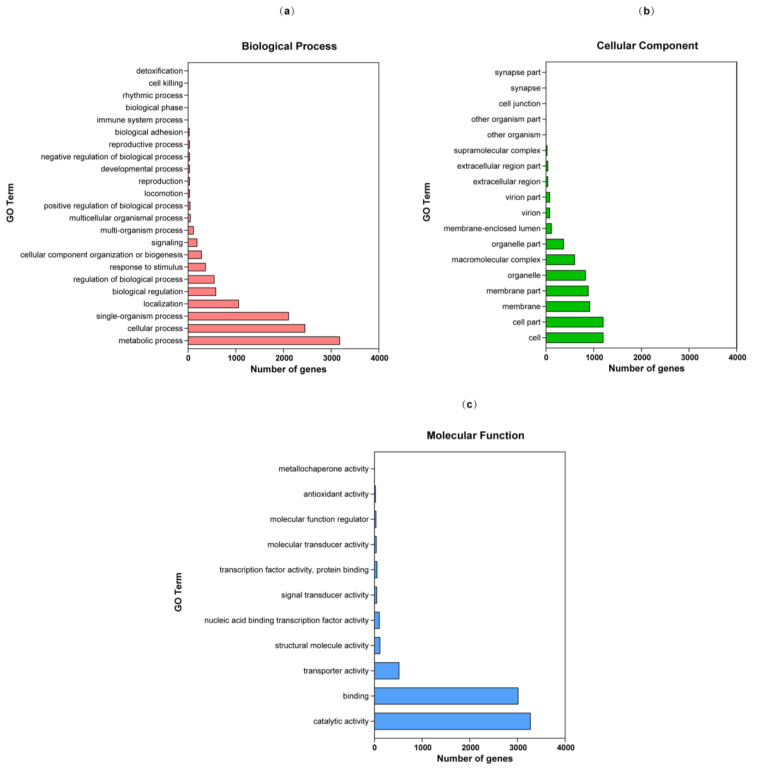
Gene Ontology (GO) functional annotation of *T. verruculosus* SJ9. (**a**) Biological Process: Biological processes accomplished through multiple molecular activities. (**b**) Cellular Component: The location of the cellular structure in which the gene product performs its function. (**c**) Molecular Function: Activity of individual gene products (including proteins and RNA) or complexes of multiple gene products at the molecular level.

Of the predicted genes, 71.33% are *T. verruculosus* SJ9 coding genes, with 5828 of them annotated in the GO database (Figure 3). The genome of *T. verruculosus* SJ9 contains 47 subclasses and three major types of protein-coding sequences: biological process (with 25 subclasses), molecular function (11 subclasses), and cellular component (18 subclasses). There were five functional classification entries with over 2000 genes identified among the three groups. The categories of these entries included binding (3019 genes), metabolic processes (3187 genes), catalytic activities (3278 genes), cellular processes (2457 genes), and single-organism processes (2112 genes).

**Figure 4:**
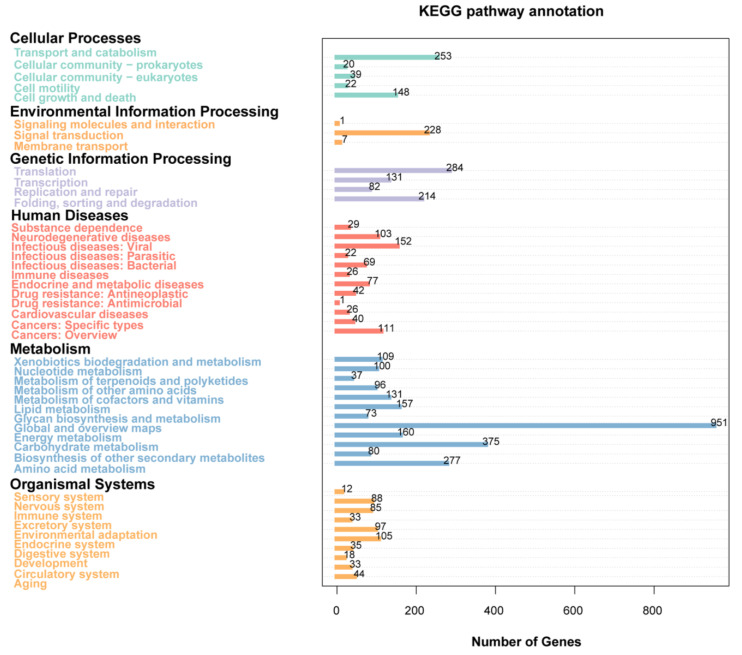
Kyoto Encyclopedia of Genes and Genomes (KEGG) functional annotation of the *T. verruculosus* SJ9 genome. The figure indicates the number of genes in the major categories and their names and associated subcategory divisions. The x-axis indicates the scale from a few genes to 1000 genes. KEGG Functions The annotations are grouped into six major categories: cellular processes, environmental information processing, genetic information, human diseases, metabolism, and organismal systems. There are 40 subcategories. Each subcategory is represented by a different color.

In order to conduct a comprehensive and systematic analysis of the metabolic pathways associated with gene products within cells, as well as the functional activities of these gene products, the gene functions of *T. verruculosus* SJ9 were annotated utilizing the KEGG database. The total number of annotated genes is statistically represented by this database (Figure 4). There were 7093 genes assigned to six major categories in KEGG: cellular processes (5 branches, *n* = 482, 6.80%), environmental information processing (3 branches, *n* = 236, 3.32%), genetic information processing (4 branches, *n* = 711, 10.02%), human diseases (12 branches, *n* = 698, 9.84%), large metabolism (12 branches, *n* = 2546, 35.89%), and organismal systems (10 branches, *n* = 550, 7.75%).

Using the CAZy database, the genomes were mapped in order to look for CAZyme existence. Based on the definitions provided in the CAZy database, a total of 562 genes, or 6.88% of all genes, could be classified into CAZymes families (Supplementary Figure S2). Oxidoreductases (AAs) (*n* = 52), glycosyltransferases (GTs) (*n* = 101), carbohydrate-binding modules (CBMs) (*n* = 74), glycosylesterases (CEs) (*n* = 27), and polysaccharide lyases (PLs) (*n* = 6) had the next greatest number of genes (*n* = 361).

TCDB contains annotations for 586 genes in total, which represents 7.17% of the expected genes (Supplementary Figure S3). Transporter genes driven by electrochemical potential accounted for 211 of them, with primary active transporters (*n* = 169), channels/pools (*n* = 65), partially characterized transport systems (*n* = 75), accessory factors involved in transport (*n* = 58), group transporters (*n* = 10), and transmembrane electron carriers (*n* = 2) following at the bottom.

**Figure 5:**
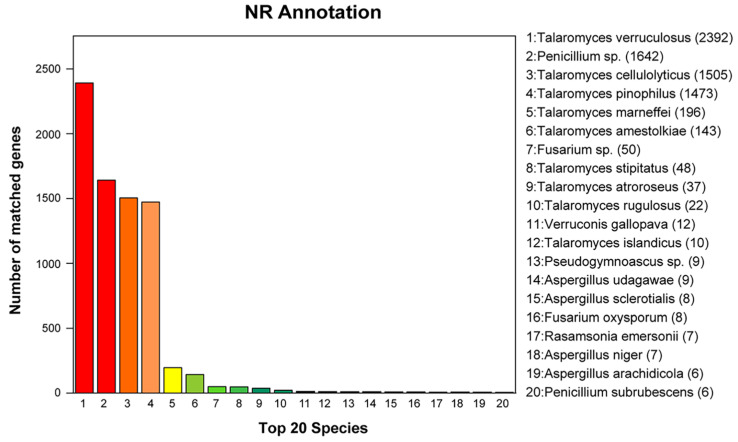
The Non-Redundant Protein Database functional annotation of the *T. verruculosus* SJ9 genome. In this figure, the x-axis represents the top 20 of 1–20 species, while the y-axis represents the number of matching genes between species on a scale of up to 2500. NR annotations were split into 20 classes. The different colors depict the top 20 species, their names, and values.

It shows a summary of the NR database annotations in Figure 5. The top 20 species that have genes that matched those identified in *T. verruculosus* SJ9 were displayed in our results. A total of 7918 *T. verruculosus* SJ9 genes, or 96.90% of all predicted genes, had hits to annotated genes in the NR database. *T. verruculosus* matched the greatest number of genes (*n* = 2392), followed by *Penicillium* sp. (*n* = 1642), *Talaromyces. cellulolyticus* (*n* = 1505), *Talaromyces. pinophilus* (*n* = 1473), and *T. marneffei* (*n* = 196). Pfam and Swiss-prot contain 5828 and 3354 annotated genes, respectively, which represent 71.33% and 41.05% of the projected genes.

#### 3.3.2. Prediction of Secreted Proteins, Secondary Metabolic Gene Clusters, and CYP450

The secreted proteins of *T. verruculosus* SJ9 are shown in Table S6. The proteins identified for secretion include 568 proteins characterized by the presence of a signal peptide, 1407 proteins displaying a transmembrane structure, and 465 proteins that contain a signal peptide but lack a transmembrane component.

It was projected which gene clusters would encode secondary metabolites. In addition to 179 genes and 19 clusters in Type I polyketide synthase (T1PKS), there were 20 genes and three clusters encoding non-ribosomal peptide synthase cluster (NRPS). Furthermore, NRPS, T1PKS contained 23 genes and two clusters, while T1PKS, Indole contained 17 genes and one cluster. Additionally, 40 genes and 8 clusters in terpene and 126 genes and 13 clusters in NRPS-like were found in our results (Supplementary Figure S5).

A superfamily of ferrous heme thiolate proteins, the CYP450 family is implicated in various physiological processes such as secondary metabolite production, xenobiotic degradation, and detoxification [60]. In Supplementary Figure S4, the annotation findings for genes related to CYP450 are displayed. It was discovered that the majority of genes (56 out of 155) encode group I E-class CYP450 proteins.

#### 3.3.3. Pathogenicity Analysis

Eight categories included the 1266 *T. verruculosus* SJ9 genes that were annotated in the PHI database (Supplementary Figure S6). The greatest number of homologs (*n* = 563) was linked to the functional class “reduced virulence.” The functional class known as “unaffected pathogenicity” has the second-highest number of genes (*n* = 393). Furthermore, the following gene classes were identified: 109 matching genes fell under the “loss of pathogenicity” class, 89 genes were classified as “NA function”, 76 similar genes were classified as “lethal gene”, and 23 genes were classified as “increased virulence (hyper-virulence)”.

The DFVF database serves as a comprehensive repository for documented issues related to fungal virulence: 2058 harmful genes from 228 fungal strains belonging to 85 taxa are present in this database, and 425 genes, or 5.20% of the expected genes in the current genomic investigation, were found in the DFVF database.

#### 3.3.4. Circular Whole-Genome Map of *T. verruculosus* SJ9

The Circos software [61] was used to display the assembled genome sequence together with the expected coding genes. It also showed the non-coding RNAs and gene prediction. A detailed circular genome diagram of *T verruculosus* SJ9 is shown in Figure S7. From the outside to the inner side of the genome, the outermost loop represents the scenario coordinates. The window (genome/1000) bp and the step size (genome/1000) bp were used to count the GC content of the genome. The dispute is represented by the purple outer area, while the blue inner part showed that the region’s total genome sequence GC content is lower than typical. The biggest deviation from the average GC content is shown by the high peak value. The following numbers were applied to the genome GC skew analysis. The algorithm used was GC/G + C. window, genome/1000 bp; step size, genome/1000 bp. The outside pink region is contradicting, whereas the innermost green piece indicates that the section’s GC level is lower than its C content. In the window genome/1000 bp and step size genome/1000 bp, the gene density quantifies the quantity of chromosome duplication and the gene thickness of coding genes, rRNA, snRNA, and tRNA (the darker the color, the higher the gene mass in the window).

### 3.4. Common Antibiotic-Degrading Enzymes Annotated to T. verruculosus SJ9

The biocatalysts known as enzymes are highly flexible and have garnered interest as a promising means of breaking down antibiotics. High selectivity and specificity, low cost, gentle operating conditions, and ease of control characterize enzymatic processes [62]. To date, many researchers have reported antibiotic-degrading enzymes. We also found many common antibiotic-degrading enzymes in the gene function annotation database of strain *T. verruculosus* SJ9 (Table 2 and Table S7).

LiP, MnP, CYP450, and Lac enzymes produced by white-rot fungi can degrade antibiotics such as TCs and FQs [20,21,22,23,63,64]. LiP is an oxidoreductase that is dependent on hydrogen peroxide. Its catalytic mechanism is as follows. The first stage is the reduction of H_2_O_2_ to water, followed by the catalytic formation of Compound I (Fe^4+^-oxo-porphyrin radical complex) and Compound II (Fe^4+^-oxo-porphyrin complex) [16]. LiP and MnP both have comparable catalytic processes, in that MnP can oxidize Mn^2+^ to Mn^3+^ chelated with organic acids, followed by oxidation reactions [16,26]. Lac, a copper-containing polyphenol oxidase, is a safer and more environmentally friendly way to catalyze reactions using molecular oxygen as an electron acceptor versus peroxidases using H_2_O_2_ [65]. There are 3, 3, 9, 1, and 122 genes involved in encoding LiP, MnP, Lac, glucanase, and CYP450 in *T. verruculosus* SJ9, respectively.

Rusch et al. found that Mycobacterium produced N-acetyltransferase, nitrate reductase, and nitrite reductase to biotransform norfloxacin, and the biotransformation products were identified as N-acetylnorfloxacin and N-nitro norfloxacin [66]. We identified 32 genes encoding N-acetyltransferases, 4 nitrate reductase genes, and 2 nitrite reductase genes in *T. verruculosus* SJ9. Park et al. used glutathione sulfotransferases (GSTs) for the degradation of tetracycline and showed that the biotoxicity of the degradation products of this enzyme was much lower than that of the parent tetracycline; the degradation rate was as high as 70% [67]. In *T. verruculosus* SJ9, one gene encodes glutathione sulfotransferase. The genes *ereA*, *ereB*, *ereC*, and *ereD* encode GST. The genes *ereA*, *ereB*, *ereC*, and *ereD* encode erythromycin esterases capable of hydrolyzing the lactone bond of macrolide antibiotics, thereby rendering the antibiotic inactive [68]. Macrolide 2′-phosphotransferase (E.C. 2.7.1.136) can phosphorylate macrolides enzymatically in order to modify them as well [69,70]. In addition, glycosylation modification of important sites for antibiotic binding to ribosomes by glycosyltransferase-catalyzed glycosylation reactions can inactivate macrolide antibiotics [28]. We identified 1 gene encoding erythromycin esterase, 1 encoding 2′-phosphotransferase, and 37 encoding glycosyltransferases in *T. verruculosus* SJ9.

We selected 13 genes from each of the common antibiotic degrading enzymes annotated to *T. verruculosus* SJ9, extracted the gene sequences and designed primers (Table S8), performed PCR on the extracted genomic DNA, and amplified all the correct bands (Figure S8).

**Table 2:** Common antibiotic-degrading enzymes annotated to *T. verruculosus* SJ9.

Degradation Enzyme	Antibiotics	Match Gene Number	References
Laccase	Fluoroquinolones, tetracyclines	9	[71,72,73]
Manganese peroxidase	Fluoroquinolones, tetracyclines	3	[74]
Glucanase	Fluoroquinolones	1	[75]
Ligninase	Fluoroquinolones, tetracyclines	3	[22,71,73]
Cytochrome P450	Fluoroquinolones	122	[73]
N-acetyltransferase	Fluoroquinolones	32	[76]
Nitrate reductase	Fluoroquinolones	4	[76]
Nitrite reductase	Fluoroquinolones	2	[66]
Glutathione S-transferase	Tetracyclines	21	[67]
Erythromycin esterase	Macrocyclic lactones	1	[77,78,79,80]
2′-phosphotransferase	Macrocyclic lactones	1	[29,30,81]
Glycosyltransferase	Macrocyclic lactones	37	[28,82,83]

### 3.5. Comparative Genomic Analysis

#### 3.5.1. Core and Pan-Genomic Analysis

**Figure 6:**
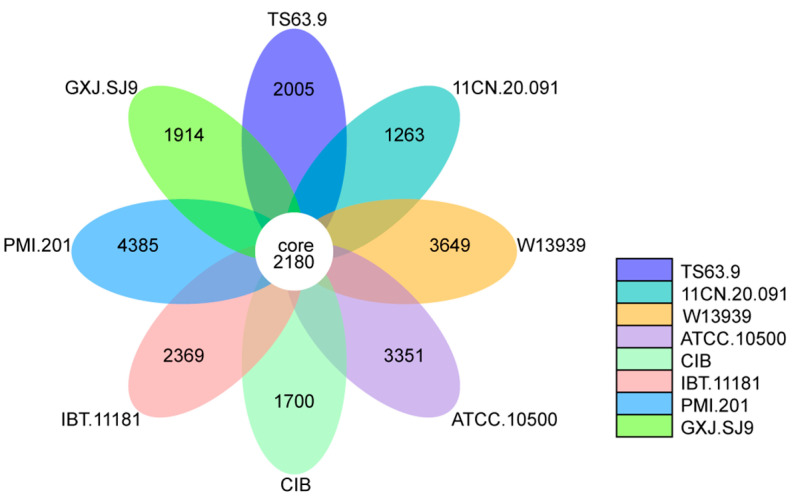
Core and pan gene orthological petals diagram of GXJ.SJ9, 11CN.20.091, W13939, ATCC.10500, CIB, IBT.11181, PMI.201, and TS63.9.

Genes that are homologous to all samples are called core genes, genes other than core genes are called dispensable genes, and genes that are only found specifically in a particular sample are called specific genes. A pan-gene collection is created by combining all shared and non-shared genes. Studying the functional differences across samples can be based on core genes and specific genes, which are likely to correlate with the similarities and features of the samples. We identified a total of 34,478 pan genes in the eight analyzed strains, comprising 2180 core genes and 32,298 dispensable genes. *T. verruculosus* TS63-9, *T. marneffei* 11CN-20-091, *T. rugulosus* W13939, *T. stipitatus* ATCC 10500, *T. amestolkiae* CIB, *T. atroroseus* IBT 11181, *T. proteolyticus* PMI_201, and *T. verruculosus* GXJ-SJ9 had species-specific gene counts of 2005, 1263, 3649, 3351, 1700, 2369, 4385, and 1914 genes, respectively (Figure 6). Through core and pan-genomic analysis, there are some potential antibiotic degradation-related enzymes that are present in all the analyzed strains (Table S7, marked in red). Meanwhile, some enzymes are only annotated in strain SJ9 (Table S7, marked in blue).

#### 3.5.2. Analysis of Species Evolution

**Figure 7:**
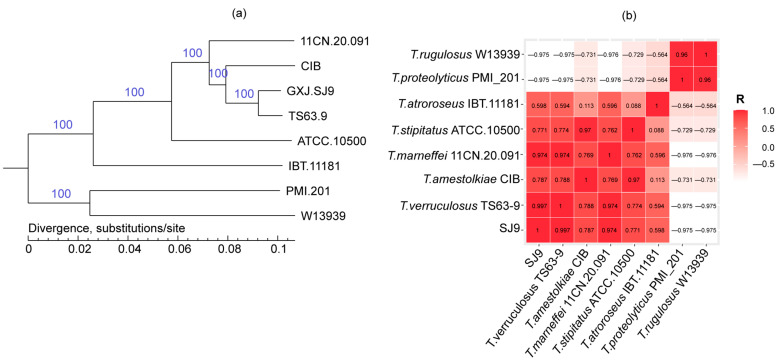
Species evolutionary analysis of SJ9 (**a**) *T. verruculosus* TS63-9, *T. marneffei* 11CN-20-091, *T. rugulosus* W13939, *T. stipitatus* ATCC 10500, *T. amestolkiae* CIB, *T. atroroseus* IBT 11181, *T. proteolyticus* PMI_201, and *T. verruculosus* GXJ-SJ9 phylogenetic tree among species. (**b**) Plot of ANI correlation coefficients for SJ9 and seven other reference genomes.

A phylogenetic tree was constructed utilizing single-copy homologous genes identified through gene family clustering, as illustrated in Figure 7a. The reliability of each branch is represented by a numerical value, with values approaching 100 signifying greater reliability. The bootstrap values for each branch were consistently 100, thereby accurately representing the evolutionary relationships among the eight strains. Additionally, the branch lengths, which are calculated based on the average number of nucleotide substitutions, serve as indicators of evolutionary distance.

There is no significant evolutionary difference between *T. verruculosus* GXJ-SJ9 and *T. verruculosus* TS63-9; rather, they are members of the same evolutionary branch. The NCBI classification database classifies *T. verruculosus* TS63-9 and *T. verruculosus* GXJ-SJ9 as the same branch of evolution, and these have no greater evolutionary distance. *T. verruculosus* GXJ-SJ9 is a member of the *T. verruculosus* species, which suggests that compared to other fungal species that belong to other taxonomic taxa, these two species are more closely related. *T. rugulosus* W13939 and *T. proteolyticus* PMI_201 have the same evolutionary branch as *T. verruculosus* GXJ-SJ9, and these have a greater evolutionary distance. The genomic reference sequences of these seven types of strains were downloaded from NCBI and used in an all-to-all ANI analysis with SJ9. The ANI correlation coefficient value between strain SJ9 and *T. verruculosus* TS63-9 was 0.997, which also suggests stronger homology between these two strains (Figure 7b).

## 4. Discussion

Members of the *Talaromyces* genus are widely distributed in nature and human environments. Some species of the genus *Talaromyces* are also producers of anti-cancer, anti-bacterial, and antifungal compounds [84,85,86,87,88]; important decomposers of lignocellulose; and producers of natural pigments [89,90]. Certain species have also been proven to be effective biocontrol agents against soil-borne pathogens. For example, *T. flavus* can inhibit verticillium wilt in tomatoes, eggplants, and potatoes [91], and degrade the cell walls of *Pythium ultimum* and *Fusarium equisetii*, which cause root rot diseases [92]. *T. pinophilus* can inhibit the activities of *Botrytis cinerea* and *Rhizoctonia solani* and reduce the probability of their parasitism on plants [93], and also has the effect of promoting the growth of rice [94].

Currently, genome information of multiple *Talaromyces* strains has been reported. In our recent study, the *T. verruculosus* SJ9 strain, isolated from strawberry rhizosphere soil, was found to be capable of effectively degrading tetracycline, enrofloxacin, and tylosin. Analysis of the SJ9 strain genome revealed a size of 40.6 Mb, an N50 scaffold length of 4,534,389 bp, and a predicted number of coding genes of 8171.

*T. verruculosus* SJ9 was identified to possess 52 AA family genes in the CAZyme database. Among them, nine genes encoding laccase (Lac) belonged to the AA1 family. Lac of the AA1 family exhibits significant catalytic activity towards aromatic compounds, which include TCs, FQs, and other non-phenolic compounds [25,95]. Some genes encoding lignin peroxidase (Lip) and manganese peroxidase (MnP) belong to the AA2 family, which are effective in removing phenolic compounds (such as TCs) and non-phenolic compounds (such as non-steroidal drugs, FQs, and β-lactams) from the environment [17,96,97]. AA1 enzyme is a multicopper oxidase that uses diols and related substances as donors, oxygen as a related substance and as a donor, and oxygen as an acceptor. The AA1 family is currently divided into three subfamilies: laccases, ferrous oxidases and laccases, and iron oxidases and laccase-like poly copper oxidases. The AA2 family contains the second group of lignin-modified peroxidases. AA2 enzymes secreting heme-containing enzymes catalyze a variety of oxidative reactions using H_2_O_2_ or organic peroxides as electron acceptors. The presence of these enzyme-encoding genes provides a strong molecular basis for the SJ9 strain in the degradation of complex organic substances. Especially in antibiotic-contaminated environments, they may alter the structure of antibiotics containing benzene rings and other structures through redox reactions, thereby achieving degradation.

Genes encoding enzymes such as CYP450, N-acetyltransferase, and GST were identified in the CYP450, Pfam, and other databases. CYP450 is generally involved in the biodegradation of antibiotics during the early stages of fungal growth [98], and N-acetylation is the most common conversion pathway for fluoroquinolones. Numerous bacterial and fungal strains are capable of catalyzing this conversion [75,99,100,101,102,103,104]. Studies have shown that the activities of antioxidant enzymes such as glutathione peroxidase, catalase, and superoxide dismutase are enhanced under antibiotic stress, which reduces antibiotic-induced oxidative stress. In addition, there was a significant increase in GST and CYP450 enzyme activity, which binds hydrogen bonds and facilitates hydrophobic interactions with antibiotics to convert them into less toxic compounds [105]. A1709 (erythromycin esterase), A1753 (2′-phosphotransferase), and 37 other genes encoding glycosyltransferases may alter the structure of macrolide antibiotics via hydrolysis, phosphorylation, and glycosylation, respectively, rendering them inactive. Overall, these antibiotic-associated enzymes provide some evidence that *T. verruculosus* SJ9 can degrade fluoroquinolone antibiotics, tetracycline antibiotics, and macrolides.

Through comparative genomic analysis, we found that the *T. verruculosus* TS63-9 had the highest genomic similarity to the SJ9 strain. This suggests a close evolutionary relationship and the likelihood of sharing similar biological functions and genetic characteristics. We also compared the genome of *T. verruculosus* TS63-9, *T. marneffei* 11CN-20-091, *T. rugulosus* W13939, *T. stipitatus* ATCC 10500, *T. amestolkiae* CIB, *T. atroroseus* IBT 11181, and *T. proteolyticus* PMI_201. This indicates both similarities and potential differences in functional capabilities compared to other strains of the same species, particularly in the specific mechanisms and capacities related to antibiotic degradation. Based on the core and pan-genomic analyses, we found that certain antibiotic-related degradation enzyme-encoding genes were specific in SJ9, while another part existed in several strains used for the analysis (Table S7). Among them, the 2′-phosphotransferase related to the degradation of macrolide lactones was only annotated in SJ9. This will help to elucidate the genetic mechanisms and potential advantages of SJ9 in antibiotic resistance, providing clues for exploring its specific degradation capabilities.

However, despite the discovery of these antibiotic-degradation-related genes in the SJ9 strain, the specific functions and synergistic mechanisms of these genes in this strain still require further in-depth investigation. For example, although genes encoding related enzymes have been identified, the activity regulation, substrate specificity, and interactions with other microorganisms or environmental factors of these enzymes in the actual environment remain unclear. Future research could employ methods such as gene expression analysis, proteomics studies, and in vitro enzyme activity assays to deeply explore the functions and regulatory mechanisms of these genes and further validate their specific roles in antibiotic degradation.

In summary, this study provides an important genomic basis for understanding the antibiotic degradation mechanism, biological functions, and potential applications of the *T. verruculosus* SJ9 strain. However, further in-depth research on its gene functions and regulatory networks is still required to fully exploit the potential of this strain in environmental governance and agricultural production.

## 5. Conclusions

In this study, a strain of *T. verruculosus* SJ9 that can degrade tetracycline, enrofloxacin, and tylosin was screened from fresh strawberry inter-root soil collected from strawberry greenhouses in Shandong Province, China. Through the correlation analysis of the whole-genome data of *T. verruculosus* SJ9, we identified a number of genes capable of encoding antibiotic-degrading enzymes in its gene function annotation database. These antibiotic-related enzymes provide some evidence that *T. verruculosus* SJ9 can degrade fluoroquinolone antibiotics, tetracycline antibiotics, and macrolide antibiotics. In summary, the complete genome sequence of *T. verruculosus* SJ9 has now been published, and this resource constitutes a significant dataset that will inform forthcoming transcriptomic, proteomic, and metabolic investigations of this fungal species. In addition, genomic studies of other filamentous fungi can utilize it as a reference. Thanks to the discoveries made in this study, the future application of this fungus in industrial production will be more rapid.

## Figures and Tables

**Table 1:** Gene assembly data of *T. verruculosus* SJ9 genome.

Parameter	Value
Contigs	30
Max_Length (bp)	8,098,124
N50_Length (bp)	4,534,389
Total length (bp)	40,609,410
GC (%)	45.4
Genome size (bp)	40,609,410
Gene number	8171
Gene total length (bp)	11,257,272
Gene average length (bp)	1378
Gene length/Genome (%)	27.72

## Data Availability

Genome sequencing data of *T. verruculosus* SJ9 generated for this study have been submitted to the NCBI (https://www.ncbi.nlm.nih.gov, accessed on: 21 March 2024). This Whole Genome Shotgun project has been deposited in GenBank under the accession number JBBVQN000000000. The raw sequencing data for the genome and the assembly reported in this paper are associated with NCBI BioProject: PRJNA1090279 and BioSample: SAMN40561778 within GenBank. The SRA accession numbers are SRR29208833 and SRR31113075.

## Supplementary Materials

The following supporting information can be downloaded at: https://www.mdpi.com/article/10.3390/genes15121643/s1, Figure S1: Summary of annotations of *T. verruculosus* SJ9; Figure S2: CAZy functional classification of *T. verruculosus* SJ9; Figure S3: TCDB functional classification of *T. verruculosus* SJ9; Figure S4: Cytochrome P450 functional classification of the *T. verruculosus* SJ9 genome; Figure S5: Secondary metabolic gene Cluster in the *T. verruculosus* SJ9 genome; Figure S6: Phenotype classification of PHI in *T. verruculosus* SJ9; Figure S7: Circular genome diagram of *T. verruculosus* SJ9. Figure S8: Photographs of PCR electrophoresis of genomic DNA of 13 antibiotic degrading enzyme genes; Table S1: Assembly summary statistics of the comparative fungal genomes; Table S2: *T. verruculosus* SJ9 genome data related to dispersed repeat sequences (DRs); Table S3: *T. verruculosus* SJ9 genome data related to tandem repeat sequences (TRs); Table S4: *T. verruculosus* SJ9 genome data related RNAs; Table S5: Summarizes of the annotations of *T. verruculosus* SJ9; Table S6: The secreted proteins of *T. verruculosus* SJ9; Table S7: Common antibiotic-degrading enzymes annotated in different databases by *T. verruculosus* SJ9; Table S8: List of primers for the antibiotic degrading enzyme gene of *T. verruculosus* SJ9.
