# Supplementary material for: Genomic Analysis of Talaromyces verruculosus SJ9: An Efficient Tetracycline-, Enrofloxacin-, and Tylosin-Degrading Fungus
Source: Genes (Basel). 2024 Dec 21;15(12):1643. doi: 10.3390/genes15121643 (PMC11675779; doi:10.3390/genes15121643)
Supplement: Supplementary file 1 [file genes-15-01643-s001.zip › Supplementary material(Fig. S1-7 and Table S4-S5).pdf]

Supplementary Material (Fig. S1-8 and Table S1-8)

1. Supplementary Figures

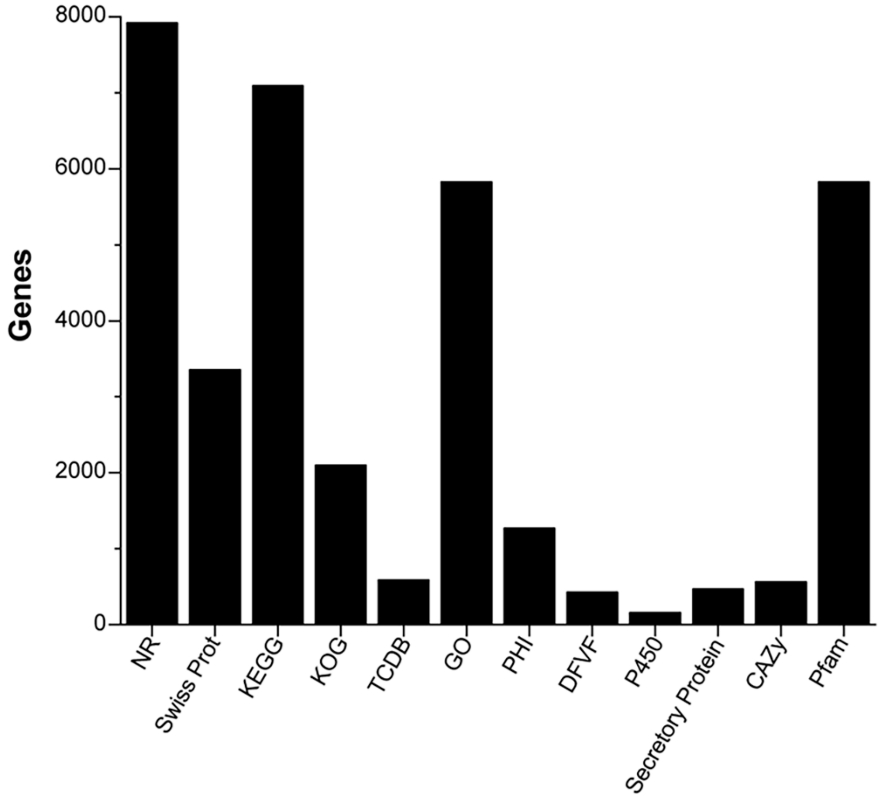

Figure S1. Summary of annotations of *Talaromyces verruculosus* SJ9.

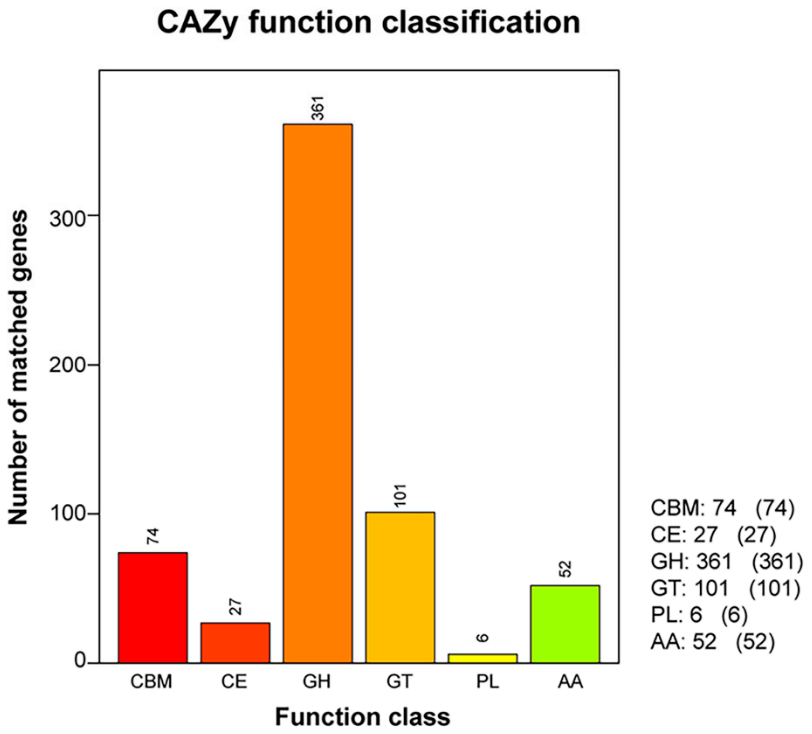

Figure S2. CAZy functional classification of *T. verruculosus* SJ9.

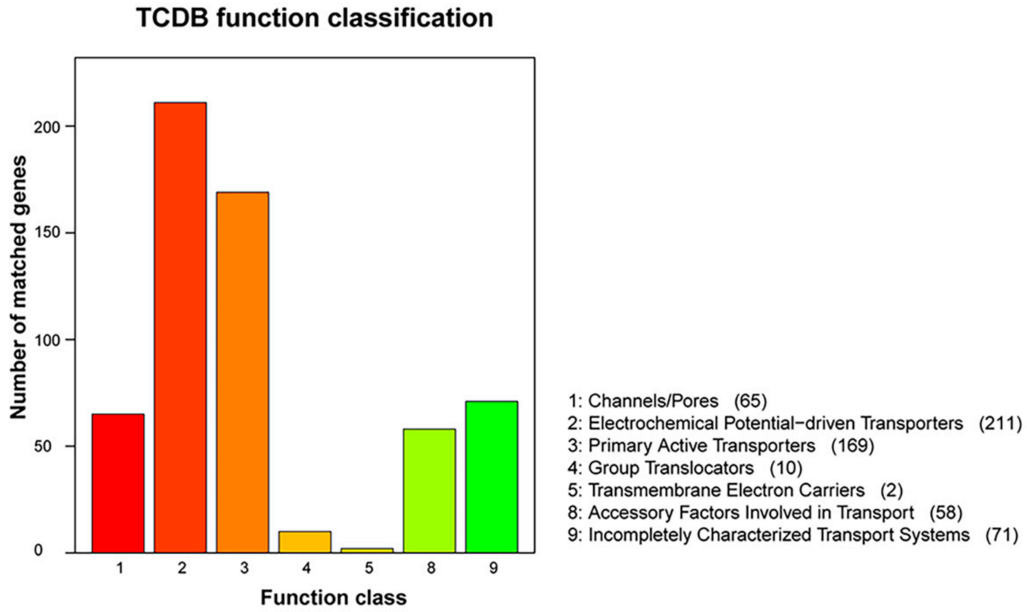

Figure S3. TCDB functional classification of *T. verruculosus* SJ9.

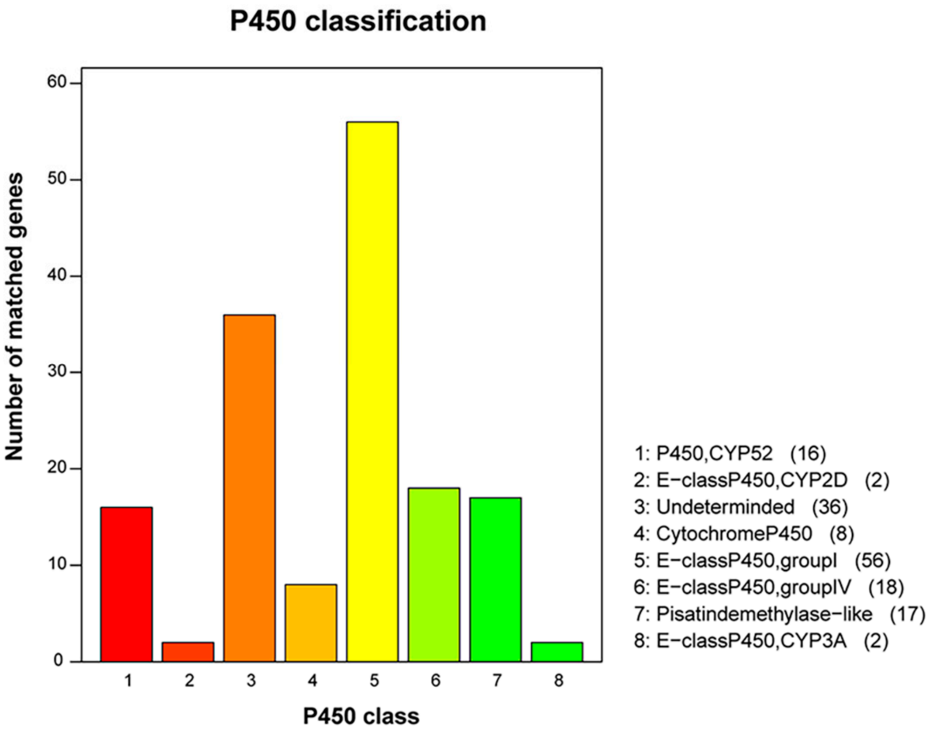

Figure S4. Cytochrome P450 functional classification of the *T. verruculosus* SJ9 genome.

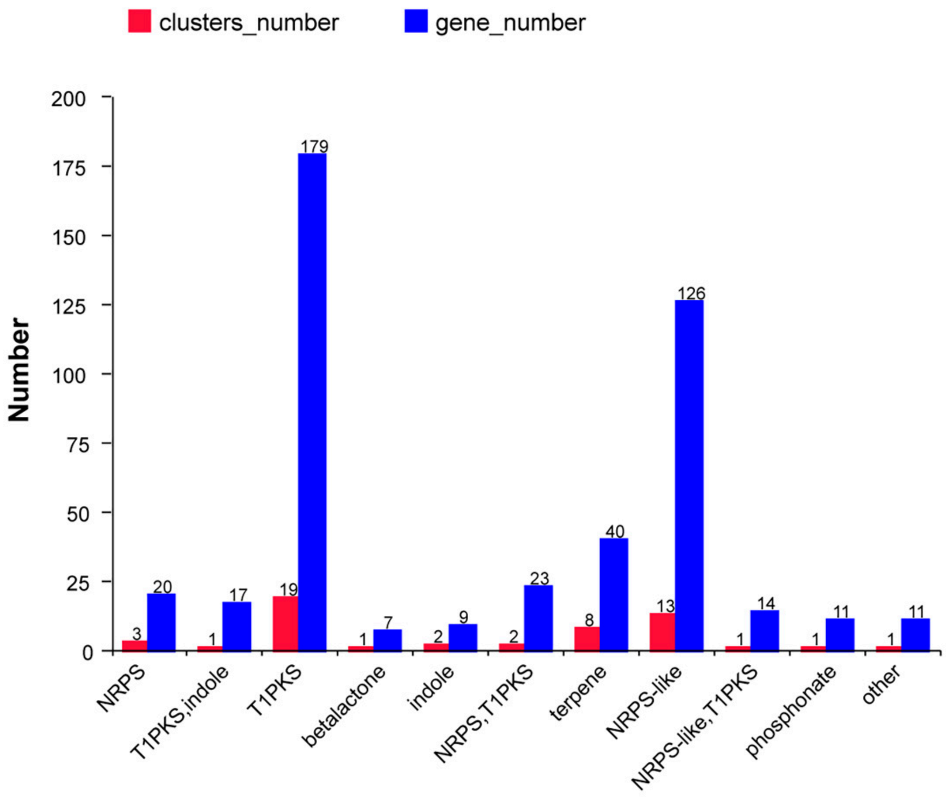

Figure S5. Secondary metabolic gene Cluster in the *T. verruculosus* SJ9 genome.

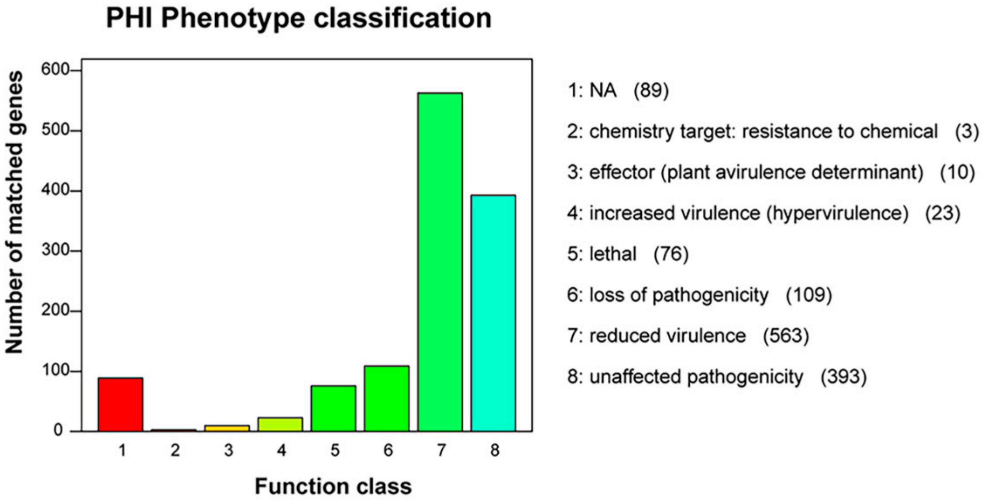

Figure S6. Phenotype classification of PHI in *T. verruculosus* SJ9.

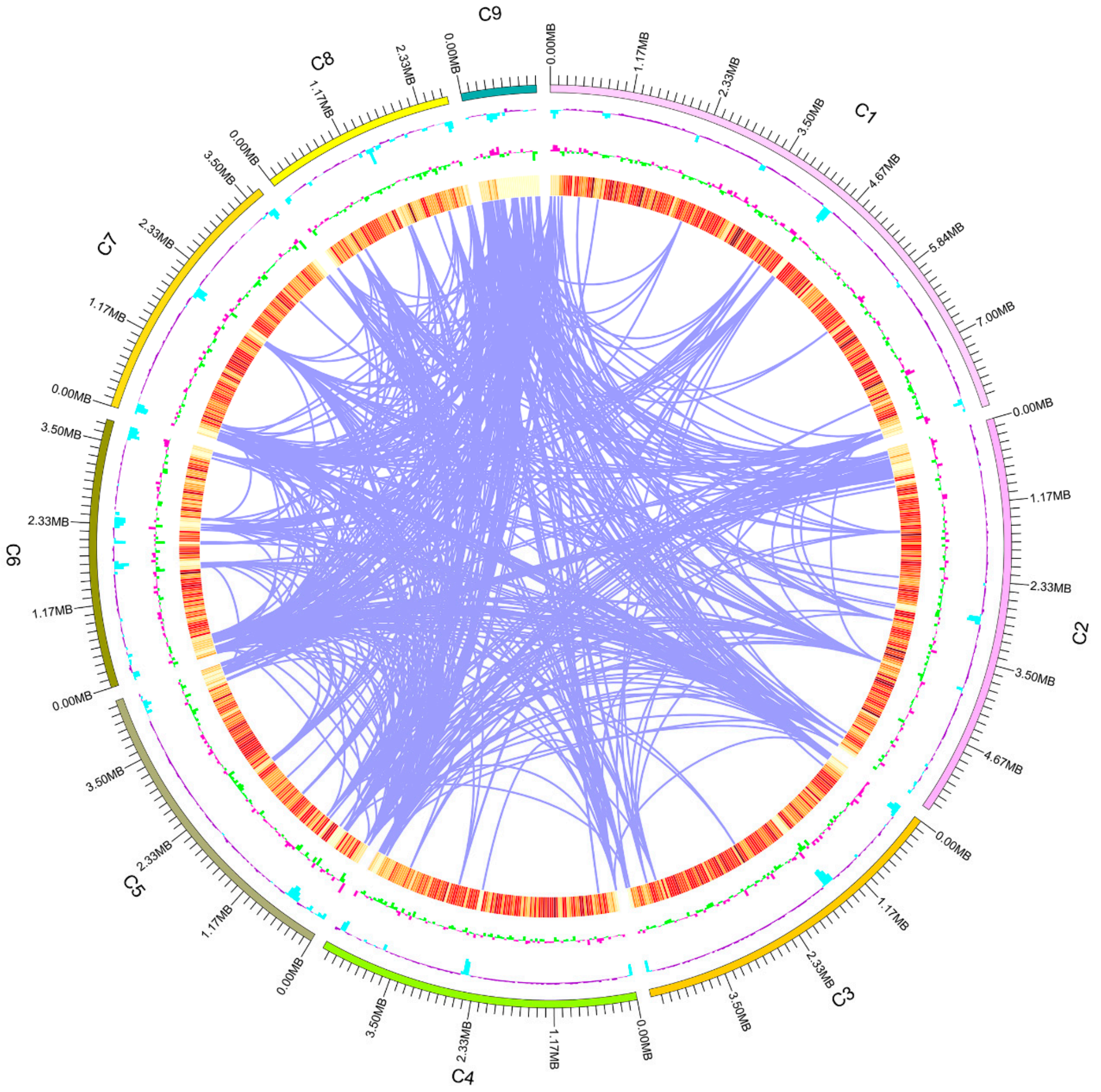

Figure S7. Circular genome diagram of *T. verruculosus* SJ9. C1 to C9 represent contig sequences exceeding 500k in the genome. From the outside to the inner side of the genome, the outermost loop represents the scenario coordinates. The window (genome/1000) bp and the step size (genome/1000) bp were used to count the GC content of the genome. The dispute is represented by the purple outer area, while the blue inner part showed that the region's total genome sequence GC content is lower than typical. The biggest deviation from the average GC content is shown by the high peak value. The following numbers were applied to the genome GC skew analysis: The algorithm used was GC/G + C. window, genome/1000 bp; step size, genome/1000 bp. The outside pink region is contradicting, whereas the innermost green piece indicates that the section's GC level is lower than its C content. In the window genome/1000 bp and step size genome/1000 bp, the gene density quantifies the quantity of chromosome du-plication and the gene thickness of coding genes, rRNA, snRNA, and tRNA (the darker the color, the higher the gene mass in the window).

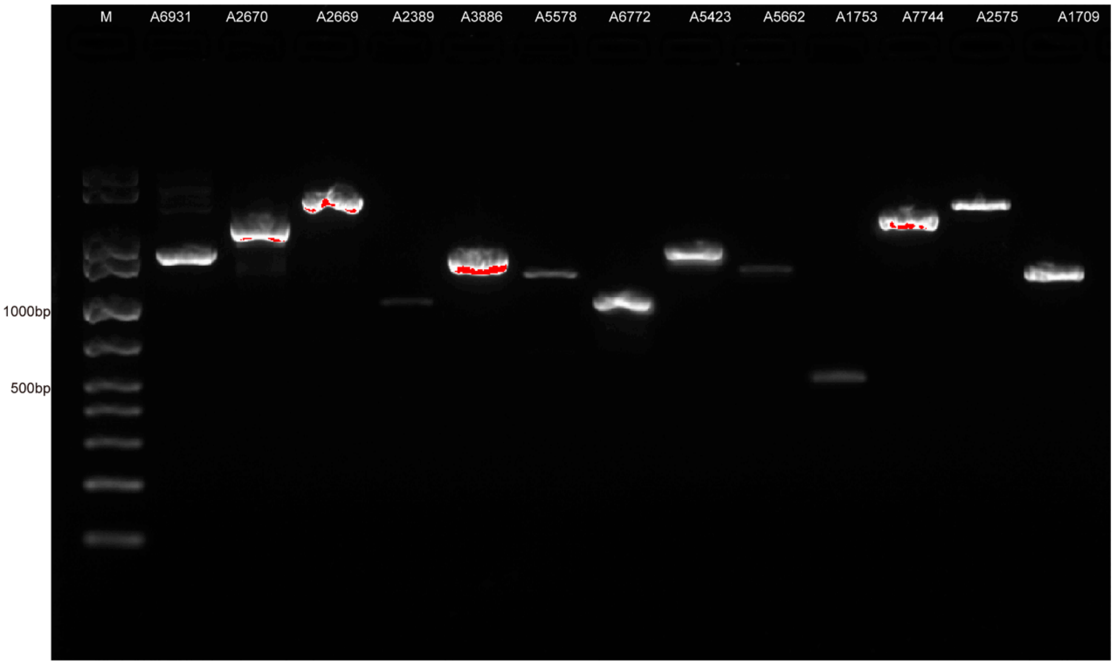

Figure S8. Photographs of PCR electrophoresis of genomic DNA of 13 antibiotic degrading enzyme genes.

2. Supplementary Tables

Table S1. Assembly summary statistics of the comparative fungal genomes.

Species	Naming	Size/Mb	GC%	N50/bp	Scaffold	Contigs	Genome Accession numbers
Talaromyces verruculosus	TS63.9	37.6	46	231,700	540	719	SAMN03944927
Talaromyces marneffei	11CN.20.091	28.2	47	3,700,000	8	8	SAMN10960796
Talaromyces rugulosus	W13939	35.8	47	5,896,210	6	6	SAMN15229550
Talaromyces stipitatus	ATCC.10500	35.7	46	897,400	820	960	SAMN02953686
Talaromyces amestolkiae	CIB	33.7	46.5	936,800	212	288	SAMN05751675
Talaromyces atroroseus	IBT.11181	30.9	44.5	376,100	48	167	SAMN03339010
Talaromyces proteolyticus	PMI.201	37.6	45.5	2,600,000	30	30	SAMN05720779
Talaromyces verruculosus	GXJ.SJ9	40.6	45.4	4,534,389	30	30	SAMN40561778

Table S2. *Talaromyces verruculosus* SJ9 genome data related to dispersed repeat sequences (DRs).

Type	Number (#)	Total Length (bp)	In Genome (%)	Average Length (bp)
LTR	1689	500,179	1.2317	299
DNA	623	125,264	0.309	207
LINE	595	183,522	0.4519	313
SINE	52	2958	0.0073	58
RC	29	2052	0.0051	71
Unknown	4	239	0.0006	60
Total	2992	808,894	1.9919	276

Table S3. *Talaromyces verruculosus* SJ9 genome data related to tandem repeat sequences (TRs).

Type	Number (#)	Repeat Size (bp)	Total Length (bp)	In Genome (%)
TR	3858	1~989	253,312	0.6238
Mini-satellite DNA	2818	10~60	154,302	0.38

Micro-satellite DNA	389	2~6	20,297	0.05

Table S4. *Talaromyces verruculosus* SJ9 genome data related RNAs.

Type	Number (#)	Average Length (bp)	Total Length (bp)
tRNA	124	100	12,402
5 s (denovo)	21	116	2436
5.8 s (denovo)	0	0	0
18 s (denovo)	1	1800	1800
28 s(denovo)	1	3460	3460
sRNA	2	266	533
snRNA	8	165	1325
miRNA	0	0	0

The specifics of Table S5-7 are in the Supplementary Material (Table S5-7).

Table S5 Summarizes of the annotations of *Talaromyces verruculosus* SJ9

Table S6 The secreted proteins of *Talaromyces verruculosus* SJ9

Table S7 Common antibiotic-degrading enzymes annotated in different databases by *Talaromyces verruculosus* SJ9

Supplementary Table 8 List of primers for the antibiotic degrading enzyme gene of *T. verruculosus* SJ9

Primer name (Gene id)	Degradation enzyme	Forward primer	Reverse primer	Length(bp)
A6931	Cytochrome P450	5'-ATGGAAAAAGGTGAGCTCTCCTAC-3'	5'-ACTAAGGGGCTTCTCGGCTG-3'	1651
A2670	Nitrate reductase	5'-ATGGCTACAGAAACGCAGGC-3'	5'-GAAAACAAGATCATTTTCATCCCA-3'	2957
A2669	Nitrite reductase	5'-AATCGATGTTATTAGCACCACCAC-3'	5'-ATGCACGAAGATCCGTCGATA-3'	3641
A2389	Glutathione S-transferase	5'-TTCATCGACTTTTACAACAAACCC-3'	5'-TAAATCATGAGTGAATATACGGGGA-3'	957
A3886	Laccase	5'-TTAACAAGATTCCCCCCAATGATC-3'	5'-ATGGCTACCCTGCAAGACGG-3'	1558
A5578	Manganese peroxidase, Ligninase	5'-CGCTGCTAGGACCTTCACCC-3'	5'-AGTCTTGAAGACATAGCGGTCCTC-3'	1198
A6772	Manganese peroxidase, Ligninase	5'-GCAGTATGAGTTGGGAGGAGATG-3'	5'-TGTACTGAAAATGCCGAAATTGA-3'	1035
A5423	Cytochrome P450	5'-GGGGCCTATCTTTCTAGCTGG-3'	5'-TTGACACCAAGTCATTATGCTCG-3'	1656
A5662	Glucanase	5'-GTTGGGGAACACGTTTGCTG-3'	5'-ATTTATCCATCCTTCAGTCGCG-3'	1272
A1753	2'-phosphotransferase	5'-AAATGCGACGGTAGTTCTTGG-3'	5'-AAAATTGTCGACACAGCTCTTTTC-3'	560
A7744	Glycosyltransferase	5'-GATGTAGACAACTACACCCGTTCAG-3'	5'-CTAAAGCAGGATGGTCATAACAGG-3'	3015
A2575	Glycosyltransferase	5'-AAGAGATCCTGTTAGCTACCCAGG-3'	5'-GATATTCGCTGCACCACCTTATC-3'	2685
A1709	Erythromycin esterase, N-acetyltransferase	5'-CTACAGTCCAAATGGATACGTCTCT-3'	5'-CCGTTGCCAGATATCAGAGATC-3'	1346
